# Preclinical Tumorigenicity Study of an Advanced Therapy Medicinal Product for Diffuse Cartilage Lesions in an Osteoarthritic Environment

**DOI:** 10.3390/cells15050429

**Published:** 2026-02-28

**Authors:** Alessandra Colombini, Vincenzo Raffo, Vincenzo Pennone, Katia Mareschi, Luciana Labanca, Laura Mangiavini, Matteo Moretti, Camilla Recordati, Federico Armando, Laura de Girolamo, Arianna B. Lovati

**Affiliations:** 1 Orthopaedic Biotechnology Lab, IRCCS Galeazzi Sant’Ambrogio Hospital, 20157 Milan, Italy; laura.degirolamo@grupposandonato.it; 2 Department of Precision and Regenerative Medicine and Ionian Area (Di.Me.Pre-J), University of Bari, 70124 Bari, Italy; v.raffo@phd.uniba.it; 3 Cell and Tissue Engineering Laboratory, IRCCS Galeazzi Sant’Ambrogio Hospital, 20157 Milan, Italy; vincenzo.pennone@grupposandonato.it (V.P.); matteo.moretti@grupposandonato.it (M.M.); arianna.lovati@grupposandonato.it (A.B.L.); 4 Stem Cell Transplantation and Cellular Therapy Laboratory, Paediatric Onco-Haematology Division, Regina Margherita Children’s Hospital, City of Health and Science of Turin, 10126 Turin, Italy; katia.mareschi@unito.it; 5 Department of Public Health and Paediatrics, University of Turin, 10126 Turin, Italy; 6 Blood Component Production and Validation Center, City of Health and Science of Turin, S. Anna Hospital, 10126 Turin, Italy; llabanca@cittadellasalute.to.it; 7 IRCCS Galeazzi Sant’Ambrogio Hospital, 20157 Milan, Italy; laura.mangiavini@unimi.it; 8 Department of Biomedical Sciences for Health, University of Milan, 20133 Milan, Italy; 9 Regenerative Medicine Division, Institute for Translational Research, Università della Svizzera Italiana (USI)–Ente Ospedaliero Cantonale (EOC), 6500 Bellinzona, Switzerland; 10 Service of Orthopaedics and Traumatology, Department of Surgery, EOC, 6900 Lugano, Switzerland; 11 Euler Institute, Faculty of Biomedical Sciences, Università della Svizzera Italiana (USI), 6900 Lugano, Switzerland; 12 Department of Veterinary Medicine and Animal Science, University of Milan, 26900 Lodi, Italy; camilla.recordati@unimi.it; 13 Mouse and Animal Pathology Laboratory, Fondazione UNIMI, 20139 Milan, Italy; 14 Department of Veterinary Sciences, University of Parma, 43126 Parma, Italy

**Keywords:** advanced therapy medicinal product (ATMP), human platelet lysate (hPL), spheroids of cartilage cells, tumorigenicity assessment, regenerative medicine/cartilage repair

## Abstract

**Highlights:**

**What are the main findings?**

- Spheroids of cartilage cells expanded at low density with human platelet lysate demonstrated genetic stability and non-tumorigenicity in vivo.
- Long-term implantation in immunodeficient mice shows no cell migration or persistence in tissues.

**What are the implications of the main findings?**

- These results support the biological safety of cartilage cell spheroids for treating cartilage lesions.
- The study provides clear preclinical evidence to support clinical translation of cell-based cartilage therapies.

**Abstract:**

Background: Advanced therapy medicinal products require rigorous preclinical testing to exclude tumorigenicity. Human articular cartilage cells expanded at low density with human platelet lysate show enhanced proliferation, matrix production, and immunomodulatory properties, supporting their use for diffuse cartilage lesions in osteoarthritic joints. This study evaluated tumorigenicity and biodistribution of cartilage cell spheroids generated using two platelet lysate sources. Methods: Cartilage cells were expanded at low density with two platelet lysates and assembled into spheroids. Cytogenetic stability was assessed by metaphase karyotyping following expansion. Immunodeficient mice received subcutaneous implantation and were monitored for 180 days. Human colon carcinoma cells and mouse fibroblasts were used as controls. Clinical follow-up, full organ histopathology, and immunohistochemistry were performed to detect human cell persistence. Results: Expanded cartilage cells showed predominantly normal karyotypes, with rare low-level mosaic chromosomal alterations not detected at the previous passage. Cartilage cell spheroids were well tolerated in vivo, with complete survival and no evidence of tumorigenicity, inflammation, or human cell persistence at implantation sites or distant organs. Control experiments confirmed the sensitivity of the model, and no systemic toxicity was observed. Conclusions: Spheroids derived from cartilage cells are non-tumorigenic, non-migratory, and biologically safe in immunodeficient mice. These findings support their development as cell-based cartilage therapies and align with regulatory recommendations for non-clinical safety evaluation.

## 1. Introduction

The development and validation of cell therapy products must comply with specific regulatory requirements, and data obtained from safety and efficacy studies of these products must be reviewed by regulatory authorities, such as the European Medicines Agency (EMA) in the European Union or the Food and Drug Administration (FDA) in the United States. In particular, advanced cell-based therapies (ATMPs) require special safety tests for authorization of clinical trials or marketing approval [1,2]. These tests include those assessing the tumorigenicity of the cells, referring to their potential to undergo malignant transformation and generate tumors, as well as their potential immunotoxicity or immunogenicity in the patient [3].

A novel cell-based therapy consisting of cartilage cell spheroids was proposed to repair diffuse cartilage lesions in an osteoarthritic environment, addressing an unmet clinical need for which current standard-of-care treatments are not effective [4]. Tumorigenicity was evaluated using a protocol previously applied to a related cell therapy [5], employing an animal xenograft model recommended for biodistribution studies of human cell-based products [6] and suitable for safety assessment of the proposed therapy.

The cell product to be tested is generated from a low-density expansion of cartilage cells cultured with human platelet lysate (hPL). hPL acts as a biological adjuvant for cell growth while preserving chondrogenic potential [7], whereas low-density culture condition is proposed as a method to enrich for cells with progenitor-like characteristics. With this strategy, we showed that a clinically relevant number of cells can be obtained from a limited amount of harvested cartilage, minimizing the need for extensive cell expansion. Beyond their remarkable chondrogenic potential, chondroprogenitor cells also demonstrated immunomodulatory activity after inflammatory priming [8], a useful feature to counteract the inflamed microenvironment typical of osteoarthritis (OA) and to promote restoration of tissue homeostasis.

Recently, data on the characterization of these cells were published and showed a higher proliferation rate compared to cells cultured at standard density, absence of replicative senescence, low immunogenicity, and preserved expression of lubricin. They also exhibited a higher expression of chondrogenic and anti-hypertrophic markers, along with greater matrix deposition than cells cultured at standard density. Cartilage cells expanded at low density induced up-regulation of CD206 and CD163 markers in macrophages—the main immune cells infiltrating arthritic joints—suggesting their ability to induce an anti-inflammatory and pro-matrix remodeling phenotype in macrophages. These findings laid the foundation for exploring the clinical utility of low-density cartilage cells to treat diffuse lesions in arthritic joints, for both autologous and allogeneic applications [9]. Spheroids derived from these cells were then produced and characterized using clinical-grade reagents, including two types of hPL.

Importantly, in line with non-clinical safety requirements [2], karyotype stability was successfully assessed during in vitro expansion before spheroid implantation.

The present study aimed to evaluate the safety of spheroids generated from low-density hPL-expanded cartilage cells for the treatment of diffuse cartilage lesions in osteoarthritic joints in an immunodeficient mouse model.

## 2. Materials and Methods

### 2.1. Isolation of Cartilage Cells and Production of Spheroids from Cartilage Cells

Knee cartilage samples were collected from four patients (three females and one male, 70–83 years old) undergoing total knee arthroplasty, after obtaining written informed consent and in accordance with a clinical protocol approved by the Ethics Committee on 16 December 2020 (n. 214/int/2020).

Cartilage cells were isolated by enzymatic digestion of cartilage and cryopreserved in human platelet lysate (hPL) containing 10% dimethyl sulfoxide (Merck, Darmstadt, Germany). Upon thawing, the cells were expanded as previously published [9], using clinical-grade reagents (all from ThermoFisher Scientific, Waltham, MA, USA) and two different hPL preparations (A and B). In particular, hPL A was a human platelet lysate manufactured by the Blood Component Production and Validation Center, City of Science and Health of Turin, S. Anna Hospital, as recently described [10], while hPL B was a commercial platelet lysate (Sexton Biotechnologies, Indianapolis, IN, USA). The isolation protocol yields a heterogeneous population of cartilage cells, including both mature chondrocytes and cartilage-derived progenitor cells.

Spheroids were produced in low-adhesion plates (PrimeSurface 96 SlitWell Plate, Twin Helix, Milan, Italy) by culturing micromasses of 200,000 cartilage cells in expansion medium for 14 days according to a previously established protocol [9].

### 2.2. Production of Spheroids from NIH/3T3 Cells

The mouse embryonic fibroblast cell line NIH/3T3 (ATCC; LGC Standards GmbH, Wesel, Germany) was used as a negative control in this study. Cells were thawed at 5 × 10^3^ cells/cm^2^ and expanded in Dulbecco’s Modified Eagle’s Medium (DMEM) containing 4500 mg/L glucose, 4 mM L-glutamine, 1 mM sodium pyruvate, and 1500 mg/L sodium bicarbonate (ATCC). To this basal medium, 10% of bovine calf serum (ATCC) and 100 U/mL penicillin, 100 μg/mL streptomycin (ThermoFisher Scientific) were added.

Spheroids were produced in low-adhesion plates (Twin Helix) by culturing micromasses of 200,000 NIH/3T3 cells in the aforementioned expansion medium for 14 days.

### 2.3. Production of Spheroids from Caco-2

The human colorectal adenocarcinoma cell line Caco-2 (ATCC) was used as positive control in this study. Cells were thawed at 20 × 10^3^ cells/cm^2^ and expanded in Dulbecco’s Modified Eagle’s Medium (DMEM) containing 4500 mg/L glucose, 1 mM sodium pyruvate, 100 U/mL penicillin, 100 μg/mL streptomycin, and 15% of fetal bovine serum (ThermoFisher Scientific).

Spheroids were produced in low-adhesion plates (Twin Helix) by culturing micromasses of 500,000 Caco-2 cells in their expansion medium for 14 days.

### 2.4. Evaluation of Karyotype Stability of Cartilage Cells

To exclude cytogenetic transformation during in vitro expansion, cartilage cells after low-density culture in hPL A and B were analyzed as previously reported [11]. Briefly, the cells were arrested in metaphase by incubation with colcemid (Invitrogen Corporation, Carlsbad, CA, USA) and then maintained in a hypotonic solution (0.075 mol/L KCl), fixed with methanol/acetic acid 3:1 (Merck) and stained with Giemsa. Cells in metaphase were analyzed with the MackType software (Nikon Corporation, Tokyo, Japan) according to the International System for Human Cytogenetic Nomenclature.

### 2.5. Histological Analysis of Spheroids from Cartilage Cells Cultured with hPL A and B

Spheroids were fixed in 10% neutral-buffered formalin (Sigma-Aldrich, St. Louis, MO, USA), rinsed with phosphate-buffered saline (PBS), and transferred to 70% ethanol. Following fixation, samples were processed for paraffin embedding and cut into 4 μm sections using a microtome. To evaluate cell morphology, sections were stained with hematoxylin and eosin (Carlo Erba Reagents, Milan, Italy), while glycosaminoglycan content was stained with Alcian Blue at pH 2.5 (Sigma-Aldrich).

For immunohistochemical detection of type I and type II collagen, sections were blocked with 2% bovine serum albumin (BSA) in PBS and subsequently incubated for 1 h at room temperature with either a rabbit monoclonal anti-collagen type I antibody (1:4000, ab138492; Abcam, Cambridge, UK) or a rabbit polyclonal anti-collagen type II antibody (1:100, ab34712; Abcam), both diluted in 5% PBS–BSA. After washing with PBS containing Tween 20, sections were incubated for 30 min with a biotinylated anti-rabbit IgG secondary antibody (1:200, VC-BA-1000-MM15; Vector Laboratories, Burlingame, CA, USA) diluted in 2% PBS–BSA, followed by a 30 min incubation with an avidin–biotin–peroxidase complex. Peroxidase activity was visualized using diaminobenzidine (Vector Laboratories) as the chromogenic substrate.

### 2.6. Mouse Model and Ethical Statement

Ethical approval for the experimental procedures was granted by the Animal Care and Use Committee (IACUC) of the Mario Negri Institute for Pharmacological Research (IRFMN) (n. 1051/2024-PR). The mice were managed following EU legislation (Directive 2010/63/EU of the European Parliament and of the Council) and Italian law (D.Lgs 26/2014). All protocols were carried out in strict compliance with the National Institutes of Health Guide for the Care and Use of Laboratory Animals, and this paper was prepared in line with the ARRIVE statement. Specifically, 29 NOD Cg-PrkdcSCIDIl2rgtm1Wjl/SzJ (NSG) female mice (20.6 ± 1.5 g; 7 weeks of age), obtained from Charles River (Calco, Lecco, Italy), were used in this study, as this immunodeficient strain is recommended for examining the biodistribution of human xenografts [5]. The mice were housed under special pathogen-free conditions in individually ventilated cages and received food and water ad libitum. After one week of acclimatization, animals were randomly allocated to the experimental groups. Group assignment was performed by an investigator not involved in subsequent surgical procedures or outcome assessment.

### 2.7. Spheroid Implantation in Mice

The animals were divided into 4 experimental groups:

1. Group hPL A: Spheroids from cartilage cells cultured in hPL A (5 spheroids/mouse, *n* = 8 mice);
2. Group hPL B: Spheroids from cartilage cells cultured in hPL B (5 spheroids/mouse, *n* = 5 mice);
3. Group Caco-2: Spheroids from Caco-2 cells, positive control for tumorigenicity assessment (20 spheroids/mouse, *n* = 8 mice);
4. Group NIH/3T3: Spheroids from NIH/3T3 cells, negative control for tumorigenicity assessment (5 spheroids/mouse, *n* = 8 mice).

Due to the nature of the implanted material (different cell types and spheroid numbers), blinding during surgical procedures was not feasible. However, post-operative clinical monitoring and body weight assessment were conducted by personnel not involved in histological evaluation and unaware of the study hypothesis.

Regardless of the type, the spheroids were implanted subcutaneously into the dorsal region of the mice, 1.5 cm caudally from the occipital pole. Under anesthesia with 1.5% isoflurane, a 0.3 cm skin incision was made bilaterally to the spine, and the spheroids were inserted using a P1000 micropipette. The wound edges were tattooed with a sterile 29-gauge needle and black tattoo liquid (Allony Dynamic Tattoo Ink, Dynamic Color Company, Pompano Beach, FL, USA) to enable precise localization of the implantation site. The skin was sutured with Ethilon 5/0 (Ethicon, Johnson & Johnson, Cincinnati, OH, USA).

The state of the surgical wound, the general condition (including monitoring for abnormal feces, signs of dehydration, abnormal neurological conditions, and any other signs of poor health), as well as water and food consumption were assessed at least once per day for seven days after implantation. Subsequently, the animals were monitored weekly regarding their general condition. In particular, animals were monitored weekly by palpation at the spheroid implantation site, assessment of general health status, and measurement of body weight.

Potential tumor growth was measured using a caliper. In the event of any symptoms that could cause suffering to the animal and were not pharmacologically treatable, euthanasia was carried out following the “humane endpoints” guidelines. These include weight loss ≥ 20%, self-inflicted injuries, and/or persistent ulcerations, fistulas, and/or local infections, tumor mass size ≥ 10 mm, and immobility > 24 h (Table 1). After 180 days, the mice were euthanized with CO_2_. After euthanasia, tumor masses and/or abnormalities at the implantation site, local lymph nodes (axillary and inguinal), and main organs (lungs, liver, gallbladder, kidneys, spleen and heart) were collected and fixed in 10% neutral buffered formalin for 4 days, then transferred to 70% ethanol for storage for subsequent histology and immunohistochemistry studies.

### 2.8. Histological Analysis of Explants

Fixed samples were routinely processed for paraffin embedding. Four μm sections from each paraffin block were stained with Hematoxylin–Eosin (HE) and evaluated under a light microscope (Leica DM750, Leica Microsystems, Wetzlar, Germany). Blinded evaluation of histological slides was carried out by CR and FA, with no information about the experimental groups. After the evaluation was completed, the codes were matched back to the original sample identities, and the data were analyzed.

For the subcutaneous implants, a general description of tumors was provided. Histological evaluation of the main morphological features of the subcutaneous tumor was performed according to the criteria reported in Table 2.

Liver, spleen, kidneys, heart, lungs, and lymph nodes (axillary and inguinal) were evaluated for the presence of metastases, as well as for tissue damage assessment.

### 2.9. Immunohistochemical Detection of Residual Human Cells

Four μm serial sections of left and right flank implants, left and right inguinal and axillary lymph nodes, liver and gallbladder, spleen, kidney, lung, and heart from animals from groups hPL A, hPL B, and Caco-2—containing human-derived cells—were stained with an anti-MHC-I antibody. Sections underwent deparaffinization and heat-induced epitope retrieval in a water bath for 30 to 40 min at 100 °C (Dewax and HIER Buffer H, Thermo Scientific Lab Vision). The slides were rinsed in PBS 1X (ThermoFisher Scientific) and placed in an autostainer (Thermo Fisher Scientific) after application of PapPen (Liquid Daido Sangyo Co., Ltd., Tokyo, Japan). Endogenous peroxidase activity was blocked by incubating sections with 3% hydrogen peroxide for 10 min. Slides were rinsed and incubated with a blocking solution containing 10% normal goat serum in PBS for 30 min at RT to prevent nonspecific background. Sections were then incubated for 1.5 h at RT with 1:600 primary monoclonal antibody anti-MHC-I (Abcam, ab52922). Sections were subsequently rinsed with PBS 1X and incubated with a biotinylated secondary antibody (Vector Laboratories) for 30 min and labeled through the avidin–biotin–peroxidase procedure for 30 min (VECTASTAIN Elite ABC-Peroxidase Kit Standard, Vector Laboratories). The immunoreaction was visualized with 3,3′-diaminobenzidine substrate after 5 min of incubation (DAB, Peroxidase DAB Substrate Kit, Vector Laboratories). Sections were counterstained with Mayer’s hematoxylin, dehydrated in a graded alcohol series, cleared in xylene, and cover-slipped with resinous mounting medium. Adequate positive controls and negative controls (omission of primary antibodies) were included in each immunolabeling assay.

### 2.10. Statistical Analyses

Sample size was determined a priori using Mead’s Resource Equation. Due to the presence of missing values resulting from animals being sacrificed at different time points, the body weight data over time were analyzed using a linear mixed-effects model rather than repeated measures ANOVA. The mixed model approach allows for the inclusion of incomplete cases under the assumption that data are missing completely at random. Comparison of survival curves among the four experimental groups was evaluated using the log-rank (Mantel–Cox) test. All statistical analyses were performed using GraphPad Prism version 8.0 (GraphPad Software, San Diego, CA, USA). Data are presented as mean ± SD.

## 3. Results

### 3.1. Karyotype Stability of Cartilage Cells

All cells expanded at low density in hPL A and hPL B exhibited predominantly nor-mal G-banded karyotypes.

A summary of all cytogenetic findings is provided in Table 3.

In one male donor (70-year-old male), a low-level sex chromosome mosaicism was observed under both hPL conditions, with two cytogenetic clones (mos 45, X [6]/46, XY [12]); importantly, at passage 1 it was reported as karyotypically normal. Similarly, an 83-year-old female donor cultured in hPL B showed a low-level autosomal mosaicism consisting of a minor trisomy 10 subclone (mos 47, XX, +10 [4]/46, XX [13]). The preceding passage was confirmed to be normal, and a repeated karyotype report described a normal 46, XX profile. This suggested that the +10 subclone was transient and did not persist upon retesting.

**Table 3:** Cytogenetic analysis of expanded cartilage cells.

Age (Years)	Sex	hPL A	hPL B
83	F	30 metaphases: 46, XX	22 metaphases: mos 47, XX, +10 [4]/46, XX [13] 20 metaphases (repeat test, passage 1): 46, XX
78	F	20 metaphases: 46, XX	20 metaphases: 46, XX
70	M	20 metaphases: mos 45, X [6]/46, XY [12]	20 metaphases: mos 45, X [6]/46, XY [12] 20 metaphases (repeat test, passage 1): mos 45, X [6]/46, XY [12]
71	F	20 metaphases: 46, XX	21 metaphases: 46, XX

Karyotypes of cells from four donors were evaluated following low-density expansion with two human platelet lysate formulations (hPL A and hPL B) at passage 2.

### 3.2. Characterization of Spheroids of Cartilage Cells Cultured with hPL A and B

**Figure 1:**
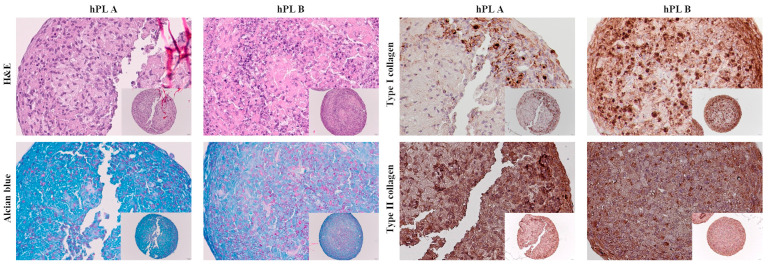
Representative images of spheroids of cartilage cells cultured with hPL A and B. H&E showed cell morphology, Alcian Blue showed glycosaminoglycan deposition, and immunohistochemistry showed type I and II collagen deposition. Insets in each panel show low-magnification images of the entire section (20×; 50 µm), while the main images display higher-magnification views of representative areas (40×; 20 µm).

Representative histological images showed differences in cellularity and extracellular matrix deposition (Figure 1). Spheroids cultured with hPL A showed fewer cells, exhibited a more abundant matrix, and showed higher glycosaminoglycan deposition in comparison with spheroids cultured with hPL B. In contrast, spheroids cultured with hPL B were richer in cells and showed greater type I collagen deposition. Immunohistochemical staining for type II collagen demonstrated its presence in spheroids cultured both with hPL A and hPL B. The images shown are representative of the observed trends; similar differences were consistently observed across multiple sections for both hPL-A and hPL-B spheroids.

### 3.3. Body Weight Monitoring and Survival Rate of Mice

**Figure 2:**
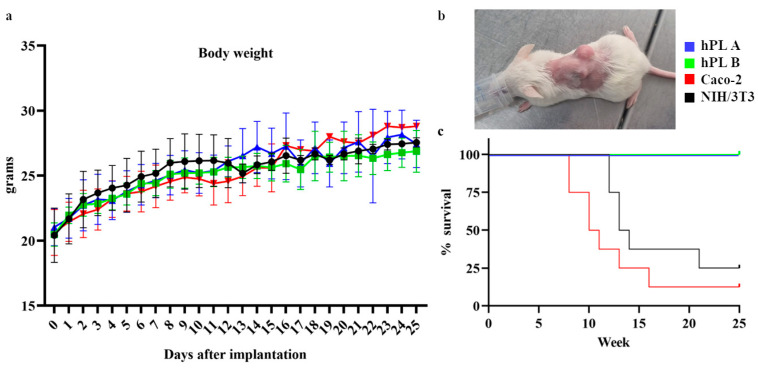
Body weight, tumorigenicity, and survival across experimental groups. Line graph of body weight over time, with group comparisons (**a**). Representative image of subcutaneous tumor formation in the Caco-2 group (**b**). Kaplan–Meier survival curve with % survival over time for the four groups (**c**).

Throughout the study duration, all murine groups maintained stable or increasing body weight, indicating good general health condition and absence of overt systemic toxicity. No individual demonstrated weight loss exceeding 20%, which is a defined humane endpoint for early euthanasia. The changes in body weight over time for each group are illustrated in Figure 2a. No statistically significant differences were observed among groups.

Both treatment groups (hPL A and hPL B) showed consistent 100% survival throughout the 25-week observation period. No mortality events were observed, suggesting a favorable safety profile with no early signs of toxicity or tumorigenesis.

In the Caco-2 group, a rapid decrease in survival started early (by week 9–11), aligning with expected tumorigenicity from this highly tumorigenic human colon carcinoma cell line (Figure 2b). As a result, tumors exceeded 1 cm in size and reached a total score of 6. This validates the sensitivity of the model with aggressive tumor growth, resulting in euthanasia of 7 subjects within week 15.

The NIH/3T3 group showed a decline in survival from week 9 onward, with 6 subjects requiring euthanasia before the planned endpoint due to subcutaneous masses ≥ 1 cm. This unexpected result suggests a potential for tumorigenicity in a murine fibroblast line that is typically used as a non-tumorigenic control.

Kaplan–Meier survival curves are presented in Figure 2c, demonstrating complete survival in both treated groups (hPL A and hPL B) compared to the survival drop observed in the Caco-2 and NIH/3T3 groups, indicating a statistically significant difference among groups (*p* < 0.0001).

### 3.4. Histological Findings

#### 3.4.1. Subcutaneous Implantation Site

**Figure 3:**
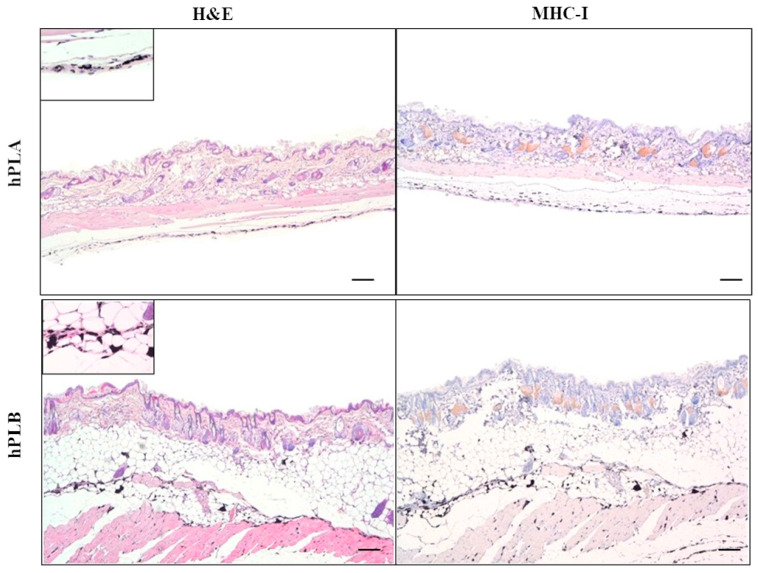
Representative image of the subcutaneous implantation site of group hPL A and hPL B. On the (**left**), H&E with normal skin and ink at the bottom. The upper left corner shows the ink-bearing cells (40×; 20 µm). On the (**right**), immunohistochemistry for MHC-I staining. Main pictures were taken at 4×; 100 µm.

In all the animals of groups hPL A and hPL B, no pathological changes were observed in the examined samples of skin and subcutis. The surrounding tissues and adnexa were normal. The presence of ink was recorded in all samples (Figure 3).

**Figure 4:**
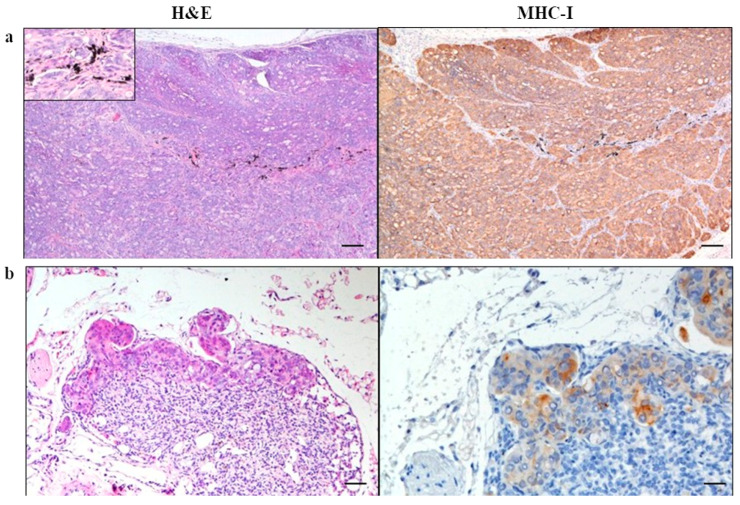
Representative image of the subcutaneous implantation site of Caco-2 group. (**a**) H&E identifies a large subcutaneous mass and ink in the middle. The upper left corner shows the ink-bearing cells (40×; 20 µm). (**b**) H&E sub-capsular sinuses expanded by tumor-implanted cells. Immunohistochemistry shows tumor cells positive for MHC-I. Pictures in (**a**) and the (**left**) picture in (**b**) were taken at 10×; 80 µm. The (**right**) picture in (**b**) was taken at 20×; 50 µm.

In the Caco-2 group, the subcutis was expanded by a non-encapsulated, well-demarcated, expansile and multifocally infiltrative, highly cellular nodular neoplasm, predominantly solid and less frequently tubular, supported by a delicate fibrovascular stroma (Figure 4).

Neoplastic cells were polygonal with indistinct cell borders, presented a high N/C ratio, and a moderate to scant amount of eosinophilic, homogeneous cytoplasm often containing variably sized, as well as clear vacuoles. Nuclei were round to oval, parabasal, with vesicular chromatin and one large, prominent nucleolus. Anisocytosis, anisokaryosis, and macrokaryosis were marked. There were numerous mitoses, often atypical. There were multifocal, variably sized areas of intra-tumoral necrosis. Some samples displayed the presence of tumor cells within vascular structures (neoplastic emboli). Five and four animals presented tumors on the right and left flanks, respectively. The average area of the tumors was 0.53 cm^2^ on the right flank, and 0.51 cm^2^ on the left one.

**Figure 5:**
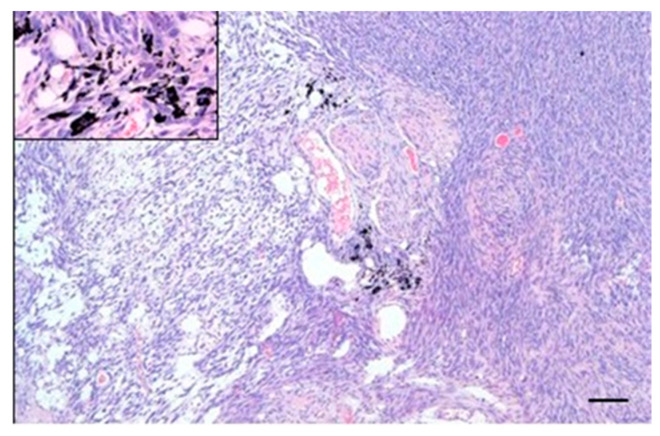
Representative image of the subcutaneous implantation site of the NIH/3T3 group. H&E shows a large subcutaneous mass and ink in the middle. The upper left corner shows a detail of the ink-bearing cells. The picture was taken at 10×; 80 µm. Close-up picture (upper left corner) was taken at 40×; 20 µm.

In the NIH/3T3 group, the subcutis was expanded by a non-encapsulated, well-demarcated, infiltrative, highly cellular nodular neoplasm, with tumor cells arranged in short or long, parallel or intersecting bundles, supported by a moderate to scant fibrous stroma (Figure 5).

Neoplastic cells were elongated with indistinct cell borders, a high N/C ratio, a scant amount of eosinophilic, fibrillar cytoplasm, and rarely contained variably sized, clear vacuoles. Nuclei were oval, central, with vesicular chromatin and one to three small, prominent nucleoli. Anisocytosis, anisokaryosis, and macrokaryosis were marked. There were numerous mitoses. There were multifocal, variably sized areas of intra-tumoral necrosis, as well as frequent multinucleated neoplastic cells with 3–5 nuclei. There were five and four animals bearing tumors on the right and left flanks, respectively. The average area of the tumors was 0.39 cm^2^ on the right flank and 0.59 cm^2^ on the left one.

#### 3.4.2. Lymph Nodes

None of the inguinal lymph nodes were found in the examined samples of mammary fat pad in any of the animal groups. When present, the axillary lymph nodes had a normal architecture and morphology. Some samples showed histiocytes with numerous intracytoplasmic black pigments (ink reabsorption). In one animal of Caco-2, sub-capsular sinuses were expanded by clusters of neoplastic cells, occasionally forming tubular structures, consistent with a metastasis of the implanted cells.

#### 3.4.3. Organs

In all evaluated samples from all groups, liver and gallbladder sections were characterized by diffuse hepatocellular cytoplasmic glycogen accumulation, and in 1–2 mice per group, there were also scattered, small foci of extramedullary myelo- and hematopoiesis. In one animal of the hPL B group, a mild, nodular hepatocellular hyperplasia was found. The gallbladder was always normal. In all evaluated samples from all groups splenic sections were characterized by diffuse extramedullary myelo- and hematopoiesis expanding the red pulp. Splenic depletion of lymphocytes in the white pulp was diffused. In addition, multifocal areas with low to moderate numbers of histiocytes with intracytoplasmic golden-brown pigment (hemosiderin, hemosiderosis) were found. There were no relevant lesions observed in the kidneys of all animals.

One animal of the hPL A group, two animals of the Caco-2 group, and one animal of the NIH/3T3 group had mild, multifocal, alveolar hemorrhages in the lungs. All other animals did not display any relevant lesions. One animal of NIH/3T3 showed a focal, small, epicardial mineralization on the left atrium. All other animals did not display any relevant heart lesions.

In the examined organs no dissemination of implanted cells or other changes related to the subcutaneous implantation were observed.

Supplementary Figure S1 shows examples of background lesions in animals from the various experimental groups.

### 3.5. Immunohistochemical Findings of Residual Human Cells at the Subcutaneous Implantation Site

No immunopositivity for MHC-I was found in the hPL A and hPL B groups, although a mild to moderate background staining on sebaceous gland cells and adipose tissue cells was observed.

In the Caco-2 group tumor cells showed a membranous immunopositivity for MHC-I, with a mild to moderate background staining on sebaceous gland cells and adipose tissue cells. Only one animal of the Caco-2 group showed metastatic cells with membranous immunopositivity for MHC-I in the axillary lymph nodes. All the other samples were negative.

No immunopositivity for MHC-I was observed in the other examined organs in any of the animal groups.

## 4. Discussion

This study aimed to investigate the tumorigenicity and potential cell migration of human cartilage cell-derived spheroids from different patients and implanted subcutaneously in immunodeficient mice for 6 months, with detailed histological and immunohistochemical analyses.

The results demonstrated that the implantation of both types of spheroids of cartilage cells, either cultured in hPL A or hPL B, was well tolerated by the mice, with no evidence of proliferative, inflammatory, or neoplastic lesions at the implantation site. Both formulations maintained comparable safety profiles, confirming that the use of two distinct GMP-grade hPLs did not affect the biological behavior of the spheroids in vivo. This comparability plays an important role in clinical translation, as it supports process robustness across a key manufacturing variable and argues against hPL-dependent induction of chromosomal abnormalities [2].

The occasional detection of ink pigment within regional lymph nodes indicated that macrophage-mediated phagocytosis and physiological pigment drainage were involved, rather than cell migration, as no human MHC-I-positive cells were observed in any of the examined lymph nodes. The limited detection of lymph nodes in the hPL A and hPL B groups likely reflects their small size in immunodeficient animals and the absence of lymphadenomegaly, a positive indicator of minimal immune reactivity to the implants and the absence of metastatic spread. The lack of proliferative or inflammatory reactions at the implantation sites is consistent with the expected biological behavior of cartilage cells, which possess limited mitotic potential and an intrinsically low oncogenic risk. This benign profile aligns with prior studies on both autologous and allogeneic chondrocyte-based constructs [5,12,14,15,16,17]. The cytogenetic data indicate that low-density expansion of cartilage cells in both hPL supplements preserves overall karyotypic stability. Nevertheless, understanding the occasional chromosomal anomalies necessitates distinguishing between culture-induced artifacts (pseudomosaicism) and true clonal expansion. In particular, the low-frequency sex chromosome mosaicism in the 70-year-old male donor is compatible with the well-known age-associated loss of chromosome Y (LOY). Regarding the transient trisomy 10 subclone in the 83-year-old female donor, it resembled a “Level II” pseudomosaicism as it was non-persistent or non-expanding over passages, supporting the interpretation of a limited event rather than progressive genomic instability [18].

In contrast, the positive control Caco-2 group, which developed rapidly growing tumors with a single case of lymph node metastatic spread, validated the sensitivity of the model.

The appearance of mesenchymal tumors in the NIH/3T3 group, despite this cell line being regarded as non-tumorigenic, is likely due to spontaneous mutations or host-derived mitogenic stimuli affecting tumorigenicity in this control line [5,13]. This may also explain the high decline in survival observed in this group, as tumor formation and rapid proliferation could compromise animal health. Further histological investigation is necessary to understand the cause, similar to the concerns raised by Zscharnack et al. [5]. These findings suggest that immortalized fibroblast lines such as NIH/3T3, while conventionally used as negative controls, may present limitations in the context of ATMP safety studies due to their genomic instability. This characteristic differs markedly from primary human cartilage cells, which exhibit limited proliferative capacity, low telomerase activity, and are non-tumorigenic. In this specific setting, the use of genomically stable primary murine cells may therefore represent a more appropriate comparator.

Overall, our results align with a previous report [5] that demonstrated that human chondrocyte spheroids implanted in NSG mice did not migrate to distant tissues or induce tumor formation during a 6-month follow-up. The present data extend this evidence by demonstrating safety using xeno-free supplements, thereby representing a clinically relevant advance for cartilage regenerative therapies. In particular, the use of hPL as a growth supplement instead of fetal bovine serum is critical, given recent studies indicating that hPL maintains genomic stability, reduces senescence markers, and preserves chondrogenic gene expression in expanded cartilage progenitors [16,19,20]. The inclusion of two independent hPL-cultured spheroid sources (hPL produced in a cell factory and the commercial formulation) provides additional robustness by confirming consistency in cellular behavior across manufacturing variables. Thus, cartilage cells expanded under low-density conditions displayed enhanced proliferation without signs of senescence or dedifferentiation, and hPL supplementation further minimizes chromosomal instability compared with xenogeneic serum [9,19,21]. Although direct data in cartilage cells are limited, studies in MSCs indicate that hPL also supports chromosomal stability compared with xenogeneic sera [22]. Finally, emerging literature has emphasized that the extracellular matrix and hypoxic microenvironment within spheroids reinforce a quiescent phenotype and suppress oncogenic pathways, distinguishing spheroid-based systems from monolayer cultures [23,24].

This study is in line with EMA (2025) and FDA (2023) guidelines on non-clinical evaluations of cell therapies, which recommend a risk-based study design that requires the use of validated immunodeficient models, appropriate follow-up periods, and sensitive human-specific detection assays such as MHC-I immunohistochemistry. In our study, the use of an NSG mouse model, long-term observation up to 180 days, and multi-organ histopathological analysis enabled the detection of delayed oncogenic transformation, confirming that cartilage cell spheroids were non-tumorigenic and did not disseminate systemically.

These results support emerging evidence that cartilage cell-based ATMPs, including spheroid or scaffold-free systems, consistently display favorable safety profiles. For instance, comparable scaffold-free systems have shown similar outcomes in both osteochondral defect and OA models [14]. Recent reports on chondrogenic spheroids in OA models [14,23] demonstrated long-term viability, matrix maintenance, and absence of neoplastic transformation, paralleling our observations. Taken together with cytogenetic data showing preserved karyotypic stability, these findings provide a coherent safety package relevant for regulatory evaluation and support further development of cartilage cell-based ATMPs under xeno-free, hPL-based manufacturing conditions.

To align with EMA expectations for investigational ATMPs, clinical translation will require a stepwise non-clinical strategy focusing on biodistribution, persistence, and tumorigenic potential. Pivotal safety studies should adhere to GLP principles where feasible, in accordance with regulatory guidance [2]. Future research should extend beyond histology to include assays for early genomic instability, DNA damage responses, or senescence signatures during in vitro expansion [21], along with efficacy assessments in OA animal models under GLP conditions.

## 5. Conclusions

These data provide a scientific basis for future regulatory-compliant studies of the proposed therapy.

For clinical translation, formal GLP-compliant tumorigenicity and biodistribution studies will be required as part of the non-clinical section of the Investigational Medicinal Product Dossier, according to EMA guidelines for ATMPs. The present results support the feasibility and safety of the proposed therapy ahead of these regulatory steps.

## Figures and Tables

**Table 1:** Post-operative surveillance clinical score.

Aspect	Score
**Body Weight**	
Sustained 15% loss for 72 h	6
Loss ≥ 20%	6
**Coat Condition**	
Piloerection	1
**Body Functions**	
Dyspnea (difficult and slow breathing)	6
**Environment**	
Diarrhea	2
Blood in stools	6
**Behavior**	
Tense and nervous when handled	1
Highly distressed by handling (e.g., agitated, vocalizing, aggressive)	3
**Locomotion/Movements**	
Abnormal posture/gait	5
Significant mobility issues	6
**Procedure-Specific Indicators**	
Tumor size: largest diameter ≥ 1 cm	6
Tumor ulceration	6
Movements hindered by the tumor	6

Score system for animal welfare assessment: 0 = No action required; 1 = Review monitoring frequency; 2–3 = Review treatment; consider additional care (e.g., veterinarian-prescribed antibiotic therapy); 4–5 = Consult the veterinarian; ≥6 = Euthanize.

**Table 2:** Criteria for histopathological evaluation of main morphological features of subcutaneous tumors.

Finding	Description
Prevalent pattern of growth	Solid; cords; lobules; glandular (tubule-acinar structures); bundles; packets, etc.
Capsule	0 = absent
	1 = present, partial
	2 = present, complete
Peripheral invasion	0 = absent
	1 = focal
	2 = multifocal
	3 = diffuse
Stroma	0 = no stroma
	1 = scant amount of stroma, mainly characterized by delicate fibrovascular septa separating groups of neoplastic cells
	2 = scant amount of stroma, mainly characterized by delicate fibrovascular septa separating groups of neoplastic cells associated with occasional broad bundles of fibrous connective tissue
	3 = moderate amount of stroma characterized by a combination of delicate fibrovascular septa and broad bundles of fibrous connective tissue separating and embedding groups of neoplastic cells
	4 = abundant stroma mainly composed of broad bundles of fibrous connective tissue separating and embedding groups of neoplastic cells
Necrosis	0 = no intra-tumoral necrotic foci
	1 = necrotic foci accounting for less than 20% of tumor extension
	2 = necrotic foci comprised between 20% and 50% of tumor extension
	3 = necrotic foci comprised between 50% and 70% of tumor extension
	4 = necrotic foci accounting for more than 70% of tumor extension
Mitoses	Number of mitoses in 3 randomly selected high-power fields (HPF = 400×)
Peritumoral inflammatory cell infiltrate	0 = absent
	1 = minimal
	2 = mild
	3 = moderate
	4 = marked

## Data Availability

The dataset supporting the conclusions of this article is available in the OSF repository, https://osf.io/jwdp9/overview?view_only=27e286b2ad694d62b87236d076c4f402, accessed on 23 December 2025.

## Supplementary Materials

The following supporting information can be downloaded at: https://www.mdpi.com/article/10.3390/cells15050429/s1, Figure S1: Representative image of background lesions across animals from different groups. All histology slides are stained with H&E. (a) In the picture there are two liver lobes and the gall bladder in the center. The upper right corner shows diffuse hepatocellular glycogen accumulation and two small foci of myelopoiesis. (b) In the picture there is a kidney and the upper right corner shows a normal histological architecture. (c) In the picture there is a spleen. The upper right corner shows splenic lymphocytes depletion, diffuse extramedullary hematopoiesis, and diffuse hemosiderosis. (d) In the picture, there is a lung with multifocal alveolar hemorrhages. (e) In the picture there is a heart. The upper right corner shows a focal epicardial mineralization. Pictures in (a,b,c,e) were taken at 4× magnification (scale bar: 100 µm). Picture in (d) was taken at 10× magnification (scale bar: 50 µm). All close-up pictures (upper right corner) were taken at 40× magnification.

## Abbreviations

The following abbreviations are used in this manuscript:

EMA	European Medicines Agency
FDA	Food and Drug Administration
ATMPs	Advanced Therapy Medicinal Products
hPL	human platelet lysate
OA	osteoarthritis
DMEM	Dulbecco’s Modified Eagle’s Medium
HE	Hematoxylin–Eosin
GMP	Good Manufacturing Practice
MHC	Major Histocompatibility Complex
MSC	Mesenchymal Stem Cells
GLP	Good Laboratory Practice
DNA	Deoxyribonucleic Acid
