# Supplementary material for: Preclinical Tumorigenicity Study of an Advanced Therapy Medicinal Product for Diffuse Cartilage Lesions in an Osteoarthritic Environment
Source: Cells. 2026 Feb 28;15(5):429. doi: 10.3390/cells15050429 (PMC12984607; doi:10.3390/cells15050429)
Supplement: Supplementary file 1 [file cells-15-00429-s001.zip › cells-4154831-supplementary.pdf]

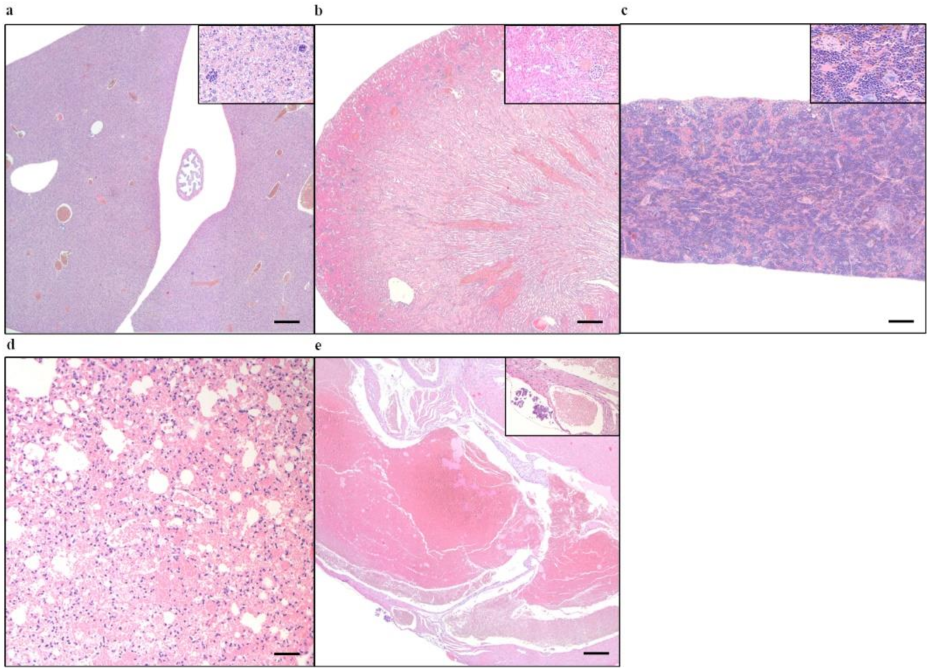

Supplementary Figure S1. Representative image of background lesions across animals from different groups. All histology slides are stained with H&E. a) In the picture there are two liver lobes and the gall bladder in the center. The upper right corner shows diffuse hepatocellular glycogen accumulation and two small foci of myelopoiesis. b) In the picture there is a kidney and the upper right corner shows a normal histological architecture. c) In the picture there is a spleen. The upper right corner shows splenic lymphocytes depletion, diffuse extramedullary hematopoiesis, and diffuse hemosiderosis. d) In the picture there is a lung with multifocal alveolar hemorrhages. e) In the picture there is a hearth. The upper right corner shows a focal epicardial mineralization. Pictures in a, b, c and e were taken at 4x magnification (scale bar: 100 μm). Picture in d was taken at 10x magnification (scale bar: 50 μm). All close-up pictures (upper right corner) were taken at 40x magnification.
